# Music Therapy Clinical Trials in Cross-Cultural Settings

**DOI:** 10.1093/geroni/igaa057.3411

**Published:** 2020-12-16

**Authors:** Tara Rose

**Affiliations:** University of Southern California, Los Angeles, California, United States

## Abstract

Music therapy in clinical trials has shown efficacy as a nonpharmacological intervention for multiple medical conditions and procedures. Every culture has music and virtually everyone on this globe enjoys music suggesting the universality of music therapy. However, in the US, most music therapy clinical trials participants are English-speaking Caucasians. That narrow pool limits our understanding of the benefits of music in an ethnically and culturally heterogeneous nation. This study looks to the international clinical trials for lessons and information that can advance U.S. studies by expanding the methodology and clinical reach to benefit a more extensive population of patients. A review of 449 studies in 48 countries from clinical trials registries supports an effort to expand music therapy studies and interventions by incorporating a cross-cultural perspective. Researchers and clinicians using international resources can increase their understanding and capacity. Globally, many standardized measures have been translated, including self-report measures of behavioral and mental health, pain, sleep, medical conditions, and symptom severity used for outcome measures, as well as music therapy measures and intervention checklists. Scientifically accepted physiological outcome measures have shown the benefits of music interventions for older adults regardless of cultural or ethnic differences. For example, neuroimaging research supports the clinically derived notion that music can address needs of people with dementia. The future will require new standards for multi-cultural research. To expand studies and methodologies, we need to include more diverse populations. This paper proposes that to do that, we must look to the global scientific community.

